# Searching for Truth: Internet Search Patterns as a Method of Investigating Online Responses to a Russian Illicit Drug Policy Debate

**DOI:** 10.2196/jmir.2270

**Published:** 2012-12-13

**Authors:** Andrey Zheluk, James A Gillespie, Casey Quinn

**Affiliations:** ^1^Menzies Centre for Health PolicySchool of Public HealthUniversity of SydneySydneyAustralia; ^2^University of NottinghamNottinghamUnited Kingdom

**Keywords:** Russia, search engine, drug dependence, policy

## Abstract

**Background:**

This is a methodological study investigating the online responses to a national debate over an important health and social problem in Russia. Russia is the largest Internet market in Europe, exceeding Germany in the absolute number of users. However, Russia is unusual in that the main search provider is not Google, but Yandex.

**Objective:**

This study had two main objectives. First, to validate Yandex search patterns against those provided by Google, and second, to test this method's adequacy for investigating online interest in a 2010 national debate over Russian illicit drug policy. We hoped to learn what search patterns and specific search terms could reveal about the relative importance and geographic distribution of interest in this debate.

**Methods:**

A national drug debate, centering on the anti-drug campaigner Egor Bychkov, was one of the main Russian domestic news events of 2010. Public interest in this episode was accompanied by increased Internet search. First, we measured the search patterns for 13 search terms related to the Bychkov episode and concurrent domestic events by extracting data from Google Insights for Search (GIFS) and Yandex WordStat (YaW). We conducted Spearman Rank Correlation of GIFS and YaW search data series. Second, we coded all 420 primary posts from Bychkov's personal blog between March 2010 and March 2012 to identify the main themes. Third, we compared GIFS and Yandex policies concerning the public release of search volume data. Finally, we established the relationship between salient drug issues and the Bychkov episode.

**Results:**

We found a consistent pattern of strong to moderate positive correlations between Google and Yandex for the terms "Egor Bychkov" (*r*_*s*_ = 0.88, *P* < .001), “Bychkov” (*r*_*s*_ = .78, *P* < .001) and “Khimki”(*r*_*s*_ = 0.92, *P* < .001). Peak search volumes for the Bychkov episode were comparable to other prominent domestic political events during 2010. Monthly search counts were 146,689 for “Bychkov” and 48,084 for “Egor Bychkov”, compared to 53,403 for “Khimki” in Yandex. We found Google potentially provides timely search results, whereas Yandex provides more accurate geographic localization. The correlation was moderate to strong between search terms representing the Bychkov episode and terms representing salient drug issues in Yandex–“illicit drug treatment” (*r*_*s*_ = .90, *P* < .001), "illicit drugs" (*r*_*s*_ = .76, *P* < .001), and "drug addiction" (*r*_*s*_ = .74, *P* < .001). Google correlations were weaker or absent–"illicit drug treatment" (*r*_*s*_ = .12, *P* = .58), “illicit drugs ” (*r*_*s*_ = -0.29, *P* = .17), and "drug addiction" (*r*_*s*_ = .68, *P* < .001).

**Conclusions:**

This study contributes to the methodological literature on the analysis of search patterns for public health. This paper investigated the relationship between Google and Yandex, and contributed to the broader methods literature by highlighting both the potential and limitations of these two search providers. We believe that Yandex Wordstat is a potentially valuable, and underused data source for researchers working on Russian-related illicit drug policy and other public health problems. The Russian Federation, with its large, geographically dispersed, and politically engaged online population presents unique opportunities for studying the evolving influence of the Internet on politics and policy, using low cost methods resilient against potential increases in censorship.

## Introduction

This is a methodological study investigating the online responses to a national debate regarding an important health and social problem in Russia. Russia has the largest Internet market in Europe, exceeding Germany in the number of users. However, Russia is unusual in that the main search provider is not Google, but Yandex. By exploring the relationship between Yandex and Google, this study contributes to the methodological literature on analysis of search patterns for public health policy.

### Theory

Studies of Internet search patterns provide a low cost, rapidly accessible data source across a range of disciplines. Underpinning these studies is the principle that each Internet search is a behavioral measure of an issue’s importance to an individual [1]. If individuals are concerned or interested in an issue, they are more likely to search for information related to that issue. The relative importance of an issue can thus be inferred from the volume of search queries for a specific term or terms representing that issue.

### Infodemiology and Infoveillance

The first studies of Internet search patterns were related to medicine. These initial studies examined the quality of online information [2], searches for cancer related information [3], and influenza surveillance use diverse data sources including Google advertisements [4], and Yahoo search trends [5]. However it was the release of Google Insights For Search (GIFS) [6] and the publication of 3 influential articles in 2009 that provided an impetus to this emerging field. The 3 influential articles are Ginsberg's study of influenza surveillance using Google data in Nature [7], Brownstein's review of online surveillance in the New England Journal of Medicine [8], and Eysenbach's consolidation of infodemiology as a distinct field of medical inquiry [9]. Eysenbach describes infodemiology as “the science of distribution and determinants of information ...specifically (on) the Internet, or in a population, with the ultimate aim to inform public health and public policy...(including) data on what people browse, buy, and read”. Where infodemiology methods are used for epidemiological surveillance, Eysenbach refers to this as infoveillance. Since 2009, Google data has been the main data source for infodemiology studies, across a wide range of health problems including dengue [10], depression [11], abortion [12], tobacco control [13], and the global Google Flu Trends site [14].

### Analysis of Search Patterns in Political Communications

Infodemiology methods have also been applied to the study of political communications and policy processes [1,2-20]. These studies are generally founded on agenda setting theory, and use Internet search patterns as a data source to complement opinion polling or traditional media (ie, television and print) coverage. Agenda setting theory suggests that issues prominently covered in traditional media are subsequently ranked as important (or salient) in public opinion polls [21,22]. The transfer of issue salience from reporting in the media to influence public opinion is an important concept in agenda setting theory [23]. Agenda setting theory appears in health advocacy studies [24], but has not yet been incorporated into studies of Internet search patterns for health policy processes.

### Agenda Setting Online

Since the early 2000s, studies of issue salience have increasingly focused on the interplay between traditional, online media, and the public agenda. A study of online bulletin board discussions found that media reports were rapidly reflected in online discussions [25]. Rather than several weeks, themes emerged in online discussions within days of traditional media reports. More recent studies have used GIFS to measure and analyse search patterns in responses to prominent media issues. Granka suggests that issue importance can be inferred from overall changes in search query volume, and that search volumes rise and fall rapidly with public interest [1]. Similarly, Scharkow suggests search patterns are the behavioral effects of salience, and provide valid and reliable measures of the public agenda [18].

### Is the Issue Salience Applicable Outside High Income Liberal Democracies?

Most studies of issue salience using search have been conducted in the United States and Organization for Economic Cooperation and Development (OECD) countries. However, there is some uncertainty as to whether the transfer of salience from traditional or online media to public opinion is universally applicable in low and middle income countries with different institutional arrangements. For example McCombs suggested that agenda setting effects require a reasonably free political system and media [26]. Other authors too, have noted the lack of research into how media shape public opinion in less-than-democratic nations. For example, Moy and colleagues point to "a glaring absence of (research about)... how citizens in these states respond to specific televised messages or their attitudes regarding certain political and social issues" [27]. This means it is difficult to infer public opinion from findings of issue salience in online media outside of high income liberal democracies.

### The State of Russian Traditional and Online Media

The Russian Federation is a middle income country with institutional arrangements that sharply contrast to those in the US or European Union (EU). Contemporary Russia has been described as a managed democracy [28] and an authoritarian state [29]. Several studies have pointed to a complex relationship between Russian traditional media, online media, and public opinion. A study of Russian's reactions to news broadcasts in 2005 reported marked differences between Russian and US viewer reactions to television news [30]. Russians were found to adopt a range of cognitive strategies, routinely reinterpreting the frames presented in television news stories using complex reasoning outcomes. These strategies were, the authors suggested, consistent with Soviet-era television viewing. Others have noted unique patterns of online media use. In 2009, Russians were the most engaged social media users globally [31]. Further, Russians engaged in unusually heterogeneous debates ranging across the political spectrum, as distinct from the partisan “echo chambers” that characterize online debate in the US [32]. These studies caution against a simple transposition of agenda setting and issue salience theories to traditional and online media. This suggests more complex process than that suggested by agenda setting and issue salience theories.

### Importance of Online Search in the Russian Federation

In 2011, Russia overtook Germany as the European country with the highest number of unique visitors online [33]. Russian Internet users grew from 43% of the population in 2010 [34] to 55% in 2012 [35]. In May 2011, Google provided 84% of Internet search queries globally [36]. The structure of the Russian-language Internet market is unique. Yandex provided 60% of Russian Internet searches in 2010-2011, compared with Google's 25% [37]. Further, Yandex offers the Wordstat (YaW) search pattern analysis tool as a direct competitor to GIFS (Table 1).

**Table 1:** Comparison of GIFS and YaW.

	GIFS	YaW
Daily data availability	2004-present for specified range	No
Weekly data availability	2004-present by default	12 months
Monthly data availability	2004-present for specified range	2 years
Time lag to availability	24 hours	8 weeks
Data display	Relative to 100% in selected date range (eg, 73% on 4 June over June - July 2011 range)	Absolute raw figure(eg, 213515)
Normalized and scaled	Yes; algorithm non-transparent	No; raw absolute values
Threshold value	Yes; algorithm non-transparent	No; raw values
Issue comparison	Yes	No
Geographic specificity	Limited	Detailed sub-regional data
Comparison concurrent terms	Yes	No
Non-English search terms	Yes	Yes

### Russian Online Media

In response to political and media constraints, Russian political debate increasingly moved online during the 2000s, using platforms such as LiveJournal [38], and more recently, Twitter [39]. RuNet has been described as a catalyst for social activism [40] political mobilization [41], as well as a channel for an alternative news agenda [42]. As Internet use grew, corruption and abuses of government power emerged as important themes online. (See for example the anticorruption blog Rospil.net [43]). By 2012, the proliferation of Russian social commentary blogs prompted a Harvard study to described Russian online media as a “transparency watchdog” [39].

RuNet's rapid growth led Russian media commentators to speculate that the Internet had eclipsed television’s traditional agenda setting function in importance [44,45]. However, other observers have cautioned against overstating the importance of Russian online media, or its distance from mainstream practices. Less optimistic observers have described pro-government blogging campaigns [46], cyber attacks [47], monitoring dissent [48], and sophisticated security filtering through SORM 2 [49]. National surveys too, suggest a more modest role for online media in shaping public opinion. A 2012 survey found 63% of Russians mostly or completely believe traditional media, while 43% mostly believe online sources [50]. In summary, as elsewhere, online media provide Russians with an information source complementing traditional media.

## Method

This is a methodological study that makes use of the unique characteristics of the Russian Internet search market. Firstly, we aimed to validate Yandex search patterns against those provided by Google. Secondly, we tested this method's adequacy for investigating online interest in a 2010 national debate over Russian illicit drug policy. In order to achieve these two aims we sought answers to the following questions:

1) What is the relationship between Google and Yandex search results?

2) What do search intensities and patterns reveal about the relative importance of an event?

3) How timely and geographically precise are GIFS and YaW results?

4) How do the search patterns during a national debate relate to salient drug issues?

### Methodological Considerations

Researchers have devoted considerable effort to establishing the validity of search pattern studies. Validity is the extent to which a test measures what it claims to measure [51]. The initial studies using GIFS established a correlation between search patterns and epidemiological surveillance data for influenza [7]. Other studies focusing on issue salience, established a correlation between search patterns, traditional media [1], and opinion polling [17]. Studies with large data sets have commonly employed ARIMA tests [52] vector auto regression and visual comparison [53], and multivariate regression [17]. Studies with smaller sample sizes have generally conducted bivariate analysis with little or no data preparation [18,54].

Several common warnings concerning validity recur in studies of search patterns. The unrepresentative demographic sampling of GIFS populations is the most frequently cited concern. The Internet user population is generally regarded as younger and wealthier than the overall population, although this cannot be elicited from search data directly [53]. Scharkow et al expand on this concern, questioning whether survey populations from traditional surveys and search pattern studies are comparable [18].

The second concern relates to the disambiguation of search terms. Individual search terms may return ambiguous search results. Care with selection of search terms, and an appropriate range of search terms is necessary to capture the breadth of potential search terms for a concept [55].

Third, several problems are associated with the limited transparency of Google's treatment of data. Google does not reveal the threshold search volume used to determine whether data is reported on a search term in GIFS [56]. This can produce unexpected zero values in time series. Further, Google provides results as relative rather than absolute data (That is, GIFS results are provided as a percentage relative to 100% during the user-defined date range-eg, 30% during June 2010). In addition, GIFS data is normalized and scaled, making comparisons between countries, regions and time spans difficult. Despite these limitations, there is a general consensus in the scholarly literature cited above that GIFS is a valid, low cost and flexible field research method. By analyzing online response to a Russian illicit drug policy episode, we hope to develop further develop the methods of search pattern analysis.

### The Bychkov Episode

Illicit drug use is a serious social and policy problem in Russia. Russian public opinion surveys have consistently rated illicit drug use among the most serious of domestic social problems [57,58]. However, Russian policy responses to this problem are generally regarded as punitive, unsupported by scientific evidence, and ineffective [59]. The punitive aspect of Russian drug policies is exemplified in 2010 by a law prohibiting dissemination of drug related health information [60]. In this complex environment, Russian reformers have compared attempts at influencing drug policy to "throwing spaghetti against a wall, and seeing what will stick” [61].

We selected the public debate surrounding the court case against socially conservative drug policy reformer Egor Bychkov as our case study. Bychkov was the president of an NGO operating a non-medical drug rehabilitation center in the provincial Urals city of Nizhny Tagil, 1900 kilometers east of Moscow. In October 2010, a local court convicted Bychkov of holding several rehabilitation clients hostage. His subsequent imprisonment sparked widespread coverage in the Russian national media. Although Bychkov was outspoken in expressing reactionary social attitudes [62] and his belief in harsh, unscientific treatment methods, he won support from socially liberal as well as conservative commentators for bringing the fight against corrupt local courts and police into the open [63]. In November 2010, following national media support, and presidential intervention, Bychkov was released on parole [64]. In June 2011, all criminal charges against Bychkov were finally annulled [65]. In 2011, having achieved national prominence, Bychkov collaborated with leading liberal bloggers and opposition politicians in Moscow [66]. In summary, the Bychkov episode was one of several concurrent episodes of opposition to Russian government policies. In each case, traditional media sparked and spread popular outrage, leaving patterns of online search.

### Questions

#### What is the Relationship between Google and Yandex Search Results?

We took the approach that this was an initial investigation with a small data set, and following earlier studies, did not cleanse data [54]. This approach had the added advantage of allowing us to quantify threshold and relative data problems in GIFS. Our data collection and analysis involved the following steps.

First, we used the terms "Bychkov" and "Egor Bychkov" to represent the Bychkov episode. We used additional terms unrelated to the Bychkov episode to provide additional context for the Bychkov episode, and to test for the validity of correlation between GIFS and YaW. We identified the main Russian domestic news events of 2010 from end-of-year compilations on government and non-government media organizations (Table 2). Further detail about this process appears below.

Second, we extracted search data series for terms representing the Bychkov episode and concurrent events from GIFS and YaW. Studies demonstrating online responses to media events typically use weekly or daily GIFS data (eg, [16]). Weekly GIFS data and monthly YaW data were available. Daily GIFS data was unavailable, and most weekly GIFS data series recorded zero values for one or more weeks during this date range. We managed this by combining the weekly GIFS ratios, including zero values, to produce a GIFS value for each month.

This produced one time series of monthly GIF values, and another of YaW monthly values. We used monthly data in the date range between March 2010 and March 2012, as these were the maximum data points available in YaW. While this produced fewer data points than some previous studies, these were sufficient to conduct a correlation analysis. We were aware monthly data was not sufficiently frequent to establish relationships between salient media issues and online search patterns.

**Figure 1:**
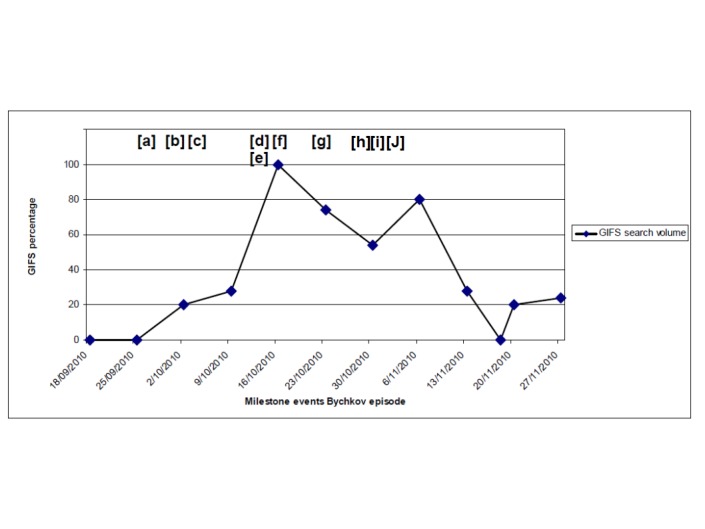
Google Insights for Search (GIFS) and milestone events in the Bychkov episode. [a] 25 September 2010 Radio Echo Moscow national broadcast about Bychkov episode 1 [11] [b] 30 September 2010 Egor Bychkov Youtube public message posted [67] [c] 2 October 2010 Radio Echo Moscow national broadcast about Bychkov episode 2 [68] [d] 12 October 2010 Nizhny Tagil Court sentences Bychkov to 3.5 year prison term [69] [e] 12 October 2010 President Medvedev publicy promises to resolve Bychkov issue [70] [f] 13 October 2010 National current affairs program dedicated to Bychkov episode [71] [g] 23 October 2010 Russian MPs debate Bychkov case on national TV [72] [h] 30 October 2010 National current affairs program dedicated to Bychkov episode [73] [I] 1 November 2010 National current affairs program dedicated to Bychkov episode [74] [j] 3 November 2010 Bychkov released from prison [75].

Third, in the absence of daily or weekly data, we corroborated events during the peak period of interest in the Bychkov episode with other available measures. We created a graph plotting GIFS daily searches against relevant media reports identified in Bychkov’s blog (Figure 1). We anticipated that the one month time range between data points would make it difficult to distinguish changes in the relationship between individual news events and increased search. We therefore turned to a weekly GIFS series to provide visual corroboration of the relationship between traditional media and search patterns over the period of maximum public and media attention to the Bychkov episode.

Fourth, we conducted bivariate analysis of GIFS and YaW data. We plotted peak monthly GIFS and YaW monthly search values to produce two time series for each search term (Table 3). Given the diversity of approaches in previous studies and limitations in available data, we chose to restrict our statistical analysis to Spearman Rank Correlations only, with the aim of establishing convergent validity between GIFS and YaW results. In the case of the GIFS time series, the zero GIFS values were artifices of censoring. This meant that variations in the GIFS searches were restricted, and therefore the correlation was biased downwards. The true correlations were likely to be stronger than the estimated statistic.

Consistent with our study aim to minimize treatment of data, we did not adjust for seasonality. Moreover, we did not assess stationarity or autocorrelation in the data, or conduct formal time-series analyses, partly because this was consistent with the study aims, and also in response to the threshold issues in the GIFS data, which would bias any attempts to stationarize the data or measure autocorrelation.

#### What do Search Patterns Reveal about the Relative Importance of an Event?

The Bychkov episode was one of a series of important domestic Russian political events in the second half of 2010. Other concurrent events included the mismanagement of nation-wide forest fires, the Khimki forest clearance protests [76], and the "blue buckets" protests against the abuse of road privileges by economic elites [77]. We identified seven protest-oriented Russian domestic events concurrent with the Bychkov episode from end-of-year compilations on government and non-government media organizations (Table 2). We selected a single term to represent each event from the terms contained in the end-of-year compilations. We used these additional search terms to provide additional context for the Bychkov episode in a complex political and media environment, and as an additional source of data with which to test the correlation between GIFS and YaW.

We then conducted limited corroboration of search patterns against opinion polls. We incorporated data from the openly accessible FOMnibus weekly national opinion poll [78] for each concurrent event. Please refer to Table 3. From FOMnibus, we used single peak values only, as an indicative measure of peak public awareness.

**Table 2:** Main events in 2010 from national Russian sources.

WCIOM ^a^	RIAN ^b^	Russia Today ^c^	Gazeta.ru ^d^
Non-government source(partially ranked)	Government source(ranked)	Government source(unranked)	Non-government source(unranked)
Forest fires	Forest fires	Moscow metro bomb	Forest fires
2018 soccer world cup	Civil society actions including Khimki forest, Egor Bychkov, blogger Kashin assault, Manezhnaya riots	START treaty	Luzhkov fired
Winter Olympics Sochi	Moscow metro bomb	Polish President killed in Smolensk plane crash	Khodorkovsky trial
Manezhnaya race riots	START treaty	65 years of Soviet Victory WW2	Manezhnaya race riots
Luzhkov fired	Administrative reforms	Forest fires	US spy scandal
	2018 soccer world cup	Luzhkov fired	Wikileaks
	Wikileaks	Khodorkovsky trial	START agreement
	US spy scandal	2018 soccer world cup	
	Police reforms		

^a^wciom.com 2010: Persons and events of the year 2010 [79]

^b^RIAN Main events of the year: fires, drought, terrorism in the metro and START 2010 [79]

^c^rt.com Russia’s ups and downs in 2010: the final cut 2010 [80]

^d^gazeta.ru Between fire and ice - main political events of the year 2010 [81]

#### How Timely and Geographically Precise are GIFS and YaW Results?

In order for search pattern data to complement existing data sources, it should offer advantages in cost, timeliness or access to data. It was the availability of valid search data in advance of traditional surveillance that initially generated interest in this field. There are several differences related to the timing and geographic precision of data released by Google and Yandex. A comparative overview of the properties of public data released by these two search providers is outlined in Table 1. In Table 3, we compared the GIFS and YaW search term results for the Bychkov episode and concurrent events for Moscow, and separately for the remainder of Russia. Importantly, neither Google nor Yandex release all their data. For example, YaW can determine the physical location of individual users to their postcode, through Internet Service Provider (ISP) hardware locations [82]. However, YaW only reveals public location data aggregated to the sub-provincial level, after two months. Google enforces similar restrictions on data availability. Commercial considerations by both search providers selling online advertising, rather than data availability, limit the release of precise and timely geographic location data.

#### How do the Search Patterns during a National Debate Relate to Salient Drug Issues?

Illicit drug use and treatment are important Russian social and policy problems. We examined search patterns in order to establish the relationship between online responses to the Bychkov episode and illicit drug issues. Firstly, we identified the two main themes associated with the Bychkov episode. These were corruption (police and judicial), and drug issues. In order to identify these high level themes, we hand coded all 420 primary posts published on Bychkov’s personal blog [83] in the date range March 2010 to March 2012. In order to identify these themes, two Russian-speaking researchers coded the primary and secondary themes in the body of each blog post, but excluded comments from readers. This resulted in 28.6% of blog posts coded as drugs (including addiction, illicit drugs, drug treatment, alcohol and tobacco), 18.3% as corruption (all sources including police and judicial) and 53.1% covering 17 other codes (including the Bychkov court case, Russian politics, the Orthodox Church, nationalism, pollution, sport, disability and philanthropic services). We assessed intercoder reliability on a random third of the total primary posts across the three themes (*Kappa* = 0.78). Consistent with the aims of this study, we then focused on the drug theme only.

Second, from Bychkov’s blog we identified the main drug themes in media reports about the Bychkov episode. We identified 57 separate national media reports referring to the Bychkov episode on Bychkov's blog within the study date range. These media reports were coded, and aggregated to two main themes, drugs and /or corruption. From the reports covering drugs, we then identified three main drug sub-themes. These were "addiction" (*narkomania*), "illicit drugs" (*narkotiki*), and "drug treatment" (*lechenie narkomanii*), which resulted in 29.2% coded for drugs (including sale and purchase), 30.1% for addiction (including use and dependence), and 39.8% for drug treatment (including medical and non-medical rehabilitation). We then assessed intercoder reliability on a random one-third sample of the total coded articles (*Kappa* = 0.75). All events referred to in Bychkov’s blog posts were corroborated using the websites of government news agency RIA-Novosti and non-government news sources.

Third, we established the relationship between search patterns for drug themes and the Bychkov episode. We did this by defining search terms, date ranges and minimizing confounding between search providers. We used the three drug themes (addiction, illicit drugs, and drug treatment) as search terms representing salient drug issues. We then investigated the relationship between these terms and the Bychkov episode, through Spearman Rank Correlations. To increase sensitivity and minimize ambiguity, we restricted search to “Egor Bychkov” to represent the Bychkov episode. Further, we restricted the date range to June 2011- June 2011. This date range search coincided with the period of Bychkov's maximum national media exposure. Finally, we conducted separate correlations within GIFS and within YaW, to prevent confounding between search providers.

## Results

We were able to gather data to achieve the two aims of this study. We gathered data with which to validate Yandex search patterns against those provided by Google. Secondly, we gathered data to test the adequacy search pattern analysis for investigating online interest in a 2010 national debate over Russian illicit drug policy. Our results are discussed in more detail below.

###  What is the Relationship between Google and Yandex Search Results?

Google and Yandex search results were positively correlated overall. We found a consistent pattern of strong to moderate positive correlations between the two search indices for the same term, both for the Bychkov episode and concurrent political events (Table 4). However, the relationship was weaker than anticipated. For example, “illicit drugs” has a weak negative relationship (*r*=-0.15). This was likely the result of GIFS returning zero values over several weeks during the specified data range. For example, GIFS searches for the term “Bychkov” displayed zero values in April, June and August 2010. During this period, YaW consistently recorded 54,000-68,000 searches per month. This is an example of the GIFS “threshold value” problem identified in earlier studies [18,65]. Within any selected date range, GIFS scales search results relative to a 100% value within that range. This scaling produces different results depending on the date range selected. This effect on GIFS data is only evident when compared with YaW data. For example, GIFS search for the term “illicit drug addiction” returned 100% in October 2010 (date range March 2010-March 2012), and 100% in November 2011 (date range November-December 2011). YaW values for these peaks GIFS dates were 132,000 and 167,102, respectively (Table 4). In summary, we were able to quantify patterns of missing data identified in earlier studies based on the use of GIFS data.

Visual examination of weekly results (Figure 1) suggests weekly peaks in GIFS indices corresponded to major milestones during the Bychkov episode. This provided further non-statistical corroboration, and face validity to the relationship between media reports and GIFS searches during the Bychkov episode.

**Table 3:** Correlation between GIFS and YaW of monthly frequency of search terms from March 2010 to March 2012.

Search item		Russian	_Correlation (rs)_	*P*	Percentage of searches originating in Moscow according to YaW
**Bychkov episode**					
	Egor Bychkov	Eгор Бычков	.88	< .001	32
	Bychkov (surname)	Бычков	.78	< .001	26
	Illicit drug addiction	Наркомания	.72	< .001	21
	Illicit drugs	Наркотики	-0.1	.64	23
	Drug addiction treatment	лечение наркомании	.53	.008	38
**Concurrent events 2010**					
	Fires	Пожары	.88	< .001	25
	Forest fires	лесные пожары	.62	< .001	20
	Khimki forest protests	Химкинский Лес	.92	< .001	65
	Yury Luzhkov(Moscow mayor Luzhkov forced resignation)	Юрий Лужков	.82	< .001	50
	Central Moscow football and race riots, and death	Манежная	.76	< .001	51
	Jailed oligarch Khodorkovsky court proceedings	Ходорковский	.8	< .001	43
	Blue buckets car protests	синие ведерки	.86	< .001	64

### What do Search Patterns Reveal about the Relative Importance of an Event?

We found search volumes for the Bychkov episode were comparable to other prominent domestic political events during 2010 (Tables 4 and 5). GIFS values provide indicative comparisons of the relative importance of an event. However, YaW provides detailed raw numbers, allowing direct comparison of search patterns for an event across regions and across time. These tables provide comparative measures of the search volumes for the Bychkov episode and other concurrent events.

**Table 4:** Peak interest in the Bychkov episode based on Public Opinion Foundation (FOM) GIFS and YaW during 2010.

Search term	Peak weekly national opinion poll - FOM - % of respondents" Which events reported in the media over the last week attracted your attention?"	Peak week GIFS (100%) (week ending)	Peak month YaW(absolute counts)
Egor Bychkov [84]	6-7 Nov 2011 <3% (bundled with criminal events and court cases)	16 Oct 2010	Oct 2010 (48,084)
Bychkov [84]	6-7 Nov 2011 <3% (bundled with criminal events and court cases)	16 Oct 2010	Oct 2010 (146,689)
Illicit drug addiction	Topic not measured in FOM	22 May 2010 and 4 Dec 2010	Nov 2010 (170,485)
Illicit drugs	Topic not measured in FOM	22 May 2010	Nov 2010 (490,026)
Drug treatment	Topic not measured in FOM	Weekly data unavailable 100% in Oct 10	Nov 10(9512)

**Table 5:** Peak interest in Russian domestic events concurrent with the Bychkov episode based on Public Opinion Foundation (FOM) GIFS and YaW during 2010.

Search term	Peak weekly national opinion poll - FOM - % of respondents "Which events reported in the media over the last week attracted your attention?"	Peak week GIFS (100%) (week ending)	Peak month YaW (absolute counts)
Fires [85]	6-7 August 2010 67% (Anomalous heat, drought, loss of the harvest, forest fires, natural catastrophes)	2 August 2010	August 2010 (22,122,660)
Forest fires [85]	6-7 August 67% as above	7 Aug 2010	August 2010 (215,397)
Manezhnaya race riots [86]	18-19 December 2010 30%	18 Dec 2010	December 2010 (408,283)
Khodorkovsky [87]	15-16 January 2011 1%	1 Nov 2011	December 2010 (199,262)
Yury Luzhkov [88]	25–26 September 2010 2%	Unavailable	September 2010 (151,743)
Khimki forest protests [89]	4-5 Sept 2010 <2% (bundled with other domestic events)	2 Oct 2010	October 2010 (53,403)
Khimki forest protests [90]	18-19 Sept 2010 <2% (bundled with other domestic events)	As above	As above
Blue buckets	Nil 0%	24 April 2010	September 2010 (39,140)

### How Timely and Geographically Precise are GIFS and YaW Results?

Google potentially provides timely search results, whereas Yandex provides more accurate geographic localization. However, both GIFS and YaW place restrictions on the data made available to the public. These restrictions are outlined in Table 1. Whereas GIFS potentially provides detailed, near real time daily data, we found gaps in actual data availability. For example, only weekly data was available for the search terms "Bychkov" and "Egor Bychkov". We described these gaps above in relation to GIFS threshold, scaling and normalization policies.

GIFS does not provide detailed subnational geographic location data in Russia. However, YaW provides disaggregated search data to the level of individual Russian provincial cities. This differentiation is important, as several of the most important domestic political events during 2010 were associated with events around the Russian capital Moscow. For example, the widely-reported Khimki forest and blue buckets protests, revealed an average of 65% and 64% of searches originating in Moscow (Table 3). This result suggests the protests were relatively more important to Moscow residents, even while Russian and international commentators ascribed national significance to these events [91,92]. By contrast, only 26% of searches for the term "Egor Bychkov" came from Moscow, and the remainder from other parts of Russia. The Bychkov episode also generated greater absolute search volumes. These two results suggest the Bychkov episode was more important across Russia than the metropolitan protests.

### How do the Search Patterns during a National Debate Relate to Salient Drug Issues?

We found moderate to strong positive correlations between search terms representing the Bychkov episode and terms representing salient drug issues in Russian media. In YaW, we found a moderate positive correlation of the term “Egor Bychkov” with the terms "illicit drugs" (*r*_*s*_ = .77), "drug addiction" (*r*_*s*_ = .74), and a strong correlation with “illicit drug treatment” (*r*_*s*_ = .90). These correlations suggest searches for “Egor Bychkov” were positively correlated to salient Russian drug issues (Table 6). GIFS produced weaker or absent correlations. We attribute this to missing data as described earlier.

**Table 6:** Relationship between Bychkov episode and substantive drug policy issues.

		Date range	*r*_*s*_	*P*
**GIFS correlations**				
	Egor Bychkov & Illicit Drugs	June 2010-June 2011	-0.29	.17
	Egor Bychkov & drug addiction	June 2010-June 2011	.68	< .001
	Egor Bychkov & illicit drug treatment	June 2010-June 2011	.12	.58
**Yaw correlations**				
	Egor Bychkov & Illicit Drugs	June 2010-June 2011	.76	< .001
	Egor Bychkov & drug addiction	June 2010-June 2011	.74	< .001
	Egor Bychkov & illicit drug treatment	June 2010-June 2011	.90	< .001

## Discussion

This study contributes to the methodological literature on the analysis of search patterns for public health policy. Firstly, we aimed to validate Yandex search patterns against those provided by Google. GIFS results have been validated against relevant offline measures across a range of scholarly domains, and across a range of countries. We were able to establish strong to moderate correlations for most search terms between GIFS and YaW. This suggests the use of YaW is a valid measure of online behavior in Russia.

We tested this method's adequacy for investigating online interest in a 2010 national debate over Russian illicit drug policy. Our use of available monthly data was insufficient to establish a statistical relationship between media reporting and search patterns for the Bychkov episode. However, we corroborated the relationship between media reporting and the Bychkov episode through the use of GIFS data within a restricted date range, opinion polling data, and media coverage. We were able to establish face validity. This suggests that media reporting influenced online behavior during the Bychkov episode. These findings are discussed in more detail below.

### Google and Yandex Search Results are Positively Correlated.

Previous studies have established the validity of GIFS data through relevant offline measures. This is the first study to validate GIFS results through YaW. We believe this approach has several advantages in the Russian-language context. By exploring the relationship between Google and Yandex search patterns in response to a drug policy debate, we were able to quantify several previously identified limitations of GIFS. We demonstrated shifting GIFS threshold values, and the extent of GIFS scaling and normalization of data through reference to YaW search results.

The presence of zero values in GIFS results merits additional discussion. The zeroes are artifices of censoring and that this means that variation in GIFS has been restricted, and therefore the correlation is biased downwards. The true correlation is likely to be stronger than the estimated statistic. The logic for the expected downward bias is that GIFS and YaW appear not to be substitutes, based upon the positive data observed.

Although time series in nature, the data were not adjusted for seasonality or non-stationarity, and autocorrelation was not assessed. This might have biased the results towards stronger, but spurious, correlation. However, this cannot currently be assessed due to the effect of the threshold used in reporting the GIFs data. If GIFS and YaW were substitutes this would bias the results in favor of a stronger negative correlation, which was not observed; if GIFS and YaW were complements (or if seasonality was strong), this would bias the results in favor of a stronger positive correlation. These issues will need to be explored in further analyses using larger and more transparent data.

At the same time, this result highlights some of the current limitations of publicly available search tools as a data source. Search pattern studies have emerged as an opportunistic response to the availability of GIFS and YaW marketing tools. The data Google and Yandex make available through these tools is only a small portion of that collected. Most of the limitations on data availability described in this paper are in fact constraints on data release imposed by the search providers themselves. Further, search providers routinely make changes to their services. For example, in September 2012, Google merged GIFS into the Trends online analysis service, incorporating GIFS capabilities into the latter [93].

### The Bychkov Episode was a Relatively Important Domestic Political Event.

Several studies have deployed GIFS to determine the importance of political episodes, as a low cost and rapid alternative to opinion polling [1,17]. We analyzed domestic events unrelated to the Bychkov episode to provide additional context for the Bychkov episode. We found search volumes for the Bychkov episode were broadly comparable to other concurrent domestic news events. For example, the controversy surrounding the jailed oligarch Mikhail Khodorkovsky was both reported in the domestic media, and produced high search volumes, suggesting this was an important issue. Conversely, the Khimki Forest and Blue Buckets protests produced low search volumes. While all three of these issues attracted international media coverage, our results suggest the last two of these were not important to Russians nationally. We did not set out to examine constraints on traditional media on Internet search. Future search pattern studies in Russia should account for the influence of mainstream media constraints on issue salience and Internet search.

### Google Potentially Provides Timely Search Results, whereas Yandex Provides More Precise Geographic Results.

Previous studies have described GIFS potential to complement existing public health data sources by providing timely, geographically precise [10,18], and otherwise inaccessible data [54]. Our results suggest timely GIFS data may not always be available in Russia. If GIFS data is missing, researchers will need to wait two months before YaW results are made available. While GIFS may not provide timely data useful for analyzing unfolding events, YaW is certain to provide delayed and detailed data. Geography is especially important in the Russian context. It is a large country, with many provincial cities, and considerable demographic variation. By comparing YaW raw data across specific regions, analysts may discern changes in search patterns for specific search terms across regions and across time. Based on our findings, we believe that YaW offers a potentially valuable tool to Russian drug policy researchers and advocates.

### The Bychkov Episode was Positively Correlated with Salient Drug Issues.

Illicit drug use has long been among one of the most important social problems troubling Russians [57]. We demonstrated a positive correlation between searches for Egor Bychkov and drug terms appearing in media reports associated with the Bychkov episode. This relationship merits further analysis. First, based on these results we are not able to distinguish between personal or sociotropic motivations for search [94]. That is, we were unable to determine whether the concurrent increase in searches for the terms "drug use", "addiction", and "drug treatment" were motivated by individual's health problems, or an interest in drug policy issues. This is consistent with Reis and Brownstein's observations concerning searches for US abortion information [12]. Second, unlike opinion polls, search patterns do not provide valency information. That is, search patterns offer no insight as to whether individuals support or oppose a specific issue. Further search terms to differentiate personal and sociotropic motivations, and to gauge valence should be considered in future search studies.

In conclusion, the Bychkov episode provides an opportunity to advance the science of search patterns. This paper investigated the relationship between Google and Yandex, and contributed to the broader methods literature by highlighting both the potential and limitations of these two search providers. We believe that YaW is a potentially valuable and underused data source for researchers working on Russian-related illicit drug policy and other public health problems. The Russian Federation, with its large, geographically dispersed, and politically engaged online population presents unique opportunities for studying the evolving influence of the Internet on politics and policy, using low cost methods resilient against potential increases in censorship. As online use grows further, primary sources of available online data will also grow. Adapting and refining research methods to best take advantage of these constantly evolving primary data sources is likely to present researchers in health policy and political communications with ongoing challenges.

## Abbreviations

EU: European Union
FOM: Public Opinion Foundation
GIFS: Google Insights For Search
NGO: Non Government Organization
OECD: Organization for Economic Cooperation and Development
RuNet: Russian Internet
SORM: 2 Russian federal government internet and telecommunications monitoring service
YaW: Yandex Wordstat
